# Preterm Birth: Effect of Corticosteroids or Immediate Cord Clamping?

**DOI:** 10.1371/journal.pmed.0030462

**Published:** 2006-10-31

**Authors:** David Hutchon, Ben Ononeze

The authors report that conventional antenatal corticosteroid therapy was received by all mothers [1]. While antenatal steroids reduce morbidity from respiratory distress syndrome and probably reduce neonatal mortality [2], there is also evidence that the steroid has an effect on brain development [3]. Multiple courses of steroids have been shown to reduce head size [4]. Multiple doses of steroids are not now recommended but may have been used before 2000. How many of these mothers received multiple courses? Furthermore, if multiple courses have a demonstrable effect, it is quite feasible that there is a minor effect on the brain by a single course.

The findings of this paper are consistent with capillary vascular damage occurring at delivery. 73% of these babies were delivered by caesarean section. We are not suggesting that the caesarean delivery in itself would have an adverse effect, but almost certainly all these babies would have had the cord clamped immediately at delivery. Clamping the cord immediately at birth, especially before the first breath is taken, interferes with the transformation from fetal to adult circulation. It is justified by the need to resuscitate the baby and maintain its temperature. The intervention of immediate cord clamping needs to be justified by evidence, which has never been sought. Indeed there is considerable evidence emerging that it is harmful to term [5], preterm [6], and very preterm infants [7]. Immediate umbilical cord clamping is the result of tradition and is carried out without thought by the vast majority of obstetricians and paediatricians. In the 1980s Peter Dunn, working in Bristol, demonstrated a technique of delivery at caesarean section for the preterm baby which avoided the hazards of immediate cord clamping [8]. This was before the use of antenatal corticosteroids or surfactant, yet the survival rates were excellent.

We would like to alert the clinical community to two issues. Firstly, antenatal corticosteroids are currently given much too readily, often when there is very little risk of preterm birth. Secondly, the cord must not be clamped immediately at delivery, especially for caesarean sections, and ways to allow resuscitation of the neonate with the cord intact must be routine clinical practice. Further research is needed to determine how much of the brain damage demonstrated in this paper could be the result of antenatal corticosteroids and immediate cord clamping.

## References

[pmed-0030462-b001] Kapellou O, Counsell SJ, Kennea N, Dyet L, Saeed N (2006). Abnormal cortical development after premature birth shown by altered allometric scaling of brain growth. PLoS Med.

[pmed-0030462-b002] Hutchon DJR (2006). Corticosteroids may not be very effective [rapid response]. eBMJ.

[pmed-0030462-b003] Thorp JA, Jones PG, Knox E, Clark RH (2002). Does antenatal corticosteroid therapy affect birth weight and head circumference?. Obstet Gynecol.

[pmed-0030462-b004] French NP, Hagan R, Evans SF, Godfrey M, Newnham JP (1999). Repeated antenatal corticosteroids: Size at birth and subsequent development. Am J Obstet Gynecol.

[pmed-0030462-b005] Ceriani Cernadas JM, Carroli G, Pellegrini L, Otano L, Ferreira M (2006). The effect of timing of cord clamping on neonatal venous hematocrit values and clinical outcome at term: A randomized, controlled trial. Pediatrics.

[pmed-0030462-b006] Rabe H, Reynolds G, Diaz-Rossello J (2006). Early versus delayed umbilical cord clamping in preterm infants (Cochrane Review).

[pmed-0030462-b007] Mercer JS, Vohr BR, McGrath MM, Padbury JF, Wallach M (2006). Delayed cord clamping in very preterm infants reduces the incidence of intraventricular hemorrhage and late-onset sepsis: A randomized, controlled trial. Pediatrics.

[pmed-0030462-b008] Dunn PM, Clinch J, Matthews T (1985). The third stage and fetal adaptation. Perinatal medicine. Proceedings of the IX European Congress of perinatal medicine held in Dublin, Ireland, 1984.

